# Untypical autoimmune pancreatitis and pancreatic cancer: differential diagnosis experiences extracted from misdiagnose of two cases

**DOI:** 10.1186/s13023-019-1217-z

**Published:** 2019-11-07

**Authors:** Gaopeng Li, Ting Liu, Jian Zheng, Wenqin Kang, Jun Xu, Zefeng Gao, Jinfeng Ma

**Affiliations:** 1 Department of general surgery, Shanxi Cancer Hospital, Shanxi Medical University, Taiyuan, Shanxi Province China; 2 Department of critical care medicine, The first hospital of Shanxi medical University, The Hospital of Shanxi University of Traditional Chinese Medicine, Taiyuan, Shanxi Province China; 3 Department of general surgery, The Hospital of Shanxi University of Traditional Chinese Medicine, Taiyuan, Shanxi Province China; 4 Department of general surgery, Shanxi Dayi Hospital, Shanxi academy of Medical science, Taiyuan, Shanxi Province China

**Keywords:** IgG4, Biopsy, Pancreatic cancer, Autoimmune pancreatitis

## Abstract

**Background:**

Differentiation between pancreatic cancer (PC) and focal form of autoimmune pancreatitis (AIP) is very challenging, with similar clinical presentations, laboratory results and morphologic imagings of US, CT, EUS, MRI, ERCP, PET-CT. Even serum IgG4 and biopsy sometimes cannot give clear-cut differential accurate diagnostis. Considering the totally different management strategy of the two diseases, accurate diagnostic value is urgently needed to remind the clinicians of the rare diagnosis of untypical AIP among frequent PC-suspected patients.

**Results:**

We present 2 laparotomy cases of AIP that had a high similar characteristic to PC and retrospectively extracted the warning signs that may help select untypical AIP in PC-suspected patients.

**Conclusions:**

We find that mild fluctuating jaundice with abdominal pain, young age, tumor marker of TPS, TPA and diverse results between variable radiological tests can help to differentiate AIP mass from PC, through retrospectively analyzing work-up process of AIP in two patients who underwent laparotomy for suspected PC.

## Background

Autoimmune pancreatitis (AIP) is a unique pancreatic manifestation of systemic immunoglobulin G4 (IgG4)-related sclerosing disease, histopathologically characterized by abundant infiltration of IgG4-positive lymphoplasmacyte and fibrosis of the pancreas with obliterative phlebitis [1]. However, contrary to typical AIPs, patients with untypical local lesion AIP and pancreatic cancer (PC) share similar clinical presentations, laboratory measurements, morphologic features of radiological examinations. To date, measurement of serum IgG4 has become a useful tool for their differentiation. However, several studies report pancreatic masses in patients with 1.6 times the upper limit of normal serum IgG4 levels (> 135 mg/dL) histopathologically proven to be PC. Moreover, quantification of serum IgG4 is often variable and inaccurate due to lack of standardization in IgG subclass assay calibration [2]. EUS-guided fine needle aspiration (FNA) may be of additional value in histological confirmation. The major limitations of the technique are operator dependence and high rate of false-negative results due to inadequate sample provided. In addition, even core biopsies will not provide enough tissue to distinguish pathological characteristics between AIP and PC, especially in PC patients with concurrent chronic pancreatitis. Also, clinically, most of PC-suspected patients cannot routinely have measurement of serum IgG4 concentrations or pre-operative histological confirmation to exclude relative rare untypical AIP. In all, as clear-cut diagnostic tool is not readily available for untypical AIP, simple diagnostic tool is urgently needed to guide the clinician in the decision-making process. Here, we present 2 laparotomy cases of AIP that had a high similar characteristic to PC and retrospectively extracted the warning signs for discrimination of untypical AIP in PC-suspected patients.

## Methods

We conducted two laparotomy cases of AIP that had a high similar characteristic to PC in Shanxi cancer hospitals in western China from June from July 2016 to August 2018. Both patients received routine treatment and care of abdominal surgery, according to medical ethics. The subjective feeling and objective data were all documented in case history. All the treatments and tests obtained informed consent of both patients.

## Results

### Case 1

A 34-year-old female visited our hospital in July 2016 because of a 4-month history of intermittent epigastralgia and poor appetite. The symptoms were not associated with food intake or daily exercise, and there was no relieving or aggravating factor. After admission, body check showed no swelling of the salivary glands and the cervical lymph nodes were not palpable. Her laboratory tests revealed elevated liver enzymes, including glutamate pyruvate transaminase (ALT) of 449 IU/L (reference range, 9–60 IU/L), glutamate oxaloacetate transaminase (AST) of 383 IU/L (reference range, 15–45 IU/L), gamma Glutamyl transpeptidase (GGT) of 823 IU/L (reference range, 10–60 IU/L, alkaline hosphate (ALP) of 1170 IU/L (reference range, 35–100 IU/L), total bilirubin (Tbil) of 183 μmol/L (reference range, 1.7–21 mg/dL), conjugated bilirubin (Dbil) of 142.1 μmol/L (reference range, 0.0–6.8 μmol/L), and unconjugated bilirubin (Ibil) of 40.9 μmol/L (reference range, 1.7–14.2 μmol/L). The results of other laboratory tests, including cholesterol profile, electrolytes, a complete blood count/differential count, renal function parameters and most tumor markers were within the normal range except high elevation of CA50, CA19–9, CA242, TPS and TPA (Table 1). Esophagogastroduodenoscopy revealed enlargement of duodenal papilla and external compression of the duodenum, which raised suspicion for a pancreatic tumor. Abdominal ultrasonography showed a mass in the uncinate process of the pancreas. Abdominal computed tomography (CT) with contrast enhancement revealed a mass arising in the end of dilated lower bile duct. Abdominal MRI depicted dilatation of the intrahepatic, extrahepatic bile ducts and main pancreatic duct caused by 5.5-cm mass lesion in the pancreatic head, with encasement of superior mesenteric vein. 18F-fluorodeoxyglucose (FDG) positron emission tomography (PET)/CT was performed and showed an FDG-avid, hypermetabolic, swollen soft tissue mass in the pancreatic head with a maximum standardized uptake value of 8.3. Adjacent low-grade FDG-avid lymph nodes with a maximum standardized uptake value of 3.0 were also noted. No FDG-avid lesions were present in the bilateral salivary glands, retroperitoneal spaces, orbiliary tracts [3]. In all, these findings were highly suggestive of obstructive jaundice due to a malignant pancreatic tumor with no distant metastasis. As the patient refuse to take US-guided biopsy and PC was highly suspected, the patient underwent pancreaticoduodenectomy and recover well. However, postoperative histologic analysis of the pancreatic head revealed moderate lymphoplasmacytic infiltration with obliterative venulitis and stromal fibrosis. Immunohistochemically, abundant IgG4-positive plasma cells (> 20/hpf and IgG4+/IgG+ plasma cell ratio > 40%) were observed infiltrating the head of the pancreas, consistent with AIP [3]. Further examination showed all the serum immune antibody including IgG4 were within normal range except slightly elevation of IgM and AMA (Table 2).

**Table 1:** Changes of tumor markers in two cases

	Case 1			Case 2			Reference
time	27 days	31 days	37 days	3 days	15 days	64 days	
CEA	1.46	0.84	0.75	1.5	0.80	0.15	<3 μ g/L
CA199	426.50↑	227.89↑	282.22↑	1.1	2.05	0.55	<20 U/mL
CA242		43.34↑	40.92↑		1.03	1.65	<12 U/mL
AFP	2.92	4.35	4.16	6.0	5.17	6.15	<15 μ g/L
CA724		4.25	1.55		0.82	0.66	<10 U/mL
CA50	222.70↑	32.44	51.52			13.55	<20 U/mL
SCC		0.24	0.27			0.19	<1 ng/mL
TPA		5.25↑	9.28↑			14.63↑	<2 ng/mL
TPS		234.57↑	765.82↑			1010.07↑	<150 U/L
VEGF		300.00	257.66			617.26	62–707 pg/mL
CA125	28					5.75	<35 U/mL
CA153	7.5						<31.3 U/mL

**Table 2:** Serological immune realted antibody: ↑ represent higher than reference, ↓ represent lower than reference. Immunoturbidimetry (ITM), Western blotting (WB), indirect immunofluorescence (IFL), blank no test

	Case 1 (postoperative)	Case 2		Reference (unit)	method
		preoperative	postoperative		
IgA	1.66	2.14	2.23	0.7–4.0 g/L	Elisa
IgG	13.34	11.34	16.3	7–16 g/L	Elisa
IgM	3.25↑	0.67	0.909	0.4–2.3 g/L	Elisa
IgE			657.50↑	20~200 IU/mL	Elisa
t-PSA			0.831	0–4 ng/mL	Elisa
f-PSA			0.301	0–4 ng/mL	Elisa
ASO			< 25.0	0–116 IU/mL	Elisa
Anti-CCP	1.75			0–25 IU/mL	Elisa
anti-TB	negative			negative	Elisa
AMA-M2	8.12			0–25 IU/mL	Elisa
Anti-a-Fodrin	negative			negative	Elisa
Complement 3	1.09		0.740↓	0.79–1.52 g/L	ITM
Complement 4	0.14		0.069↓	0.1–0.4 g/L	ITM
RF	1.95		73.1↑	<20 IU/mL	ITM
IgG1			1170.0↑	405–1011 mg/dL	ITM
IgG2			234	169–768 mg/dL	ITM
IgG3			32.8	11–85 mg/dL	ITM
IgG4	5.43	98	266.0↑	<201 mg/dL	ITM
ANA	negative	negative		negative	IFL
AKA	negative			negative	IFL
ASMA	negative			negative	IFL
AMA	1:100 ↑			negative	IFL
AMA-M2	negative	negative		negative	WB
Sp100	negative	negative		negative	WB
LKM1	negative	negative		negative	WB
Gp210	negative	negative		negative	WB
LC1	negative	negative		negative	WB
SLA	negative	negative		negative	WB
Anti-Nucleosomes	negative			negative	WB
Anti-dsDNA	negative			negative	WB
SmD1	negative			negative	WB
Anti-PO	negative			negative	WB
Anti-Histones	negative			negative	WB
U1-SnRNP	negative			negative	WB
Anti-SSA/Ro60	negative			negative	WB
Anti-SSA/Ro52	negative			negative	WB
SSB/La	negative			negative	WB
Anti-Slc-70	negative			negative	WB
Anti-CENP-B	negative			negative	WB
Anti-Jo-1	negative			negative	WB

### Case 2

A 49-year-old male was admitted to our hospital in August 2018 complaining of epigastralgia and jaundice. He had no history of swollen salivary glands and the cervical lymph nodes were not palpable. Laboratory data included: ALT of 57 IU/L (reference range, 9–60 IU/L), AST of 42 IU/L (reference range, 15–45 IU/L), GGT of 133 IU/L (reference range, 10–60 IU/L, ALP of 191 IU/L (reference range, 455–125 IU/L), Tbil of 147.4 μmol/L (reference range, 1.7–21 mg/dL), Dbil of 1.5.6 μmol/L (reference range, 0.0–6.8 μmol/L), and Ibil of 41.8 μmol/L (reference range, 1.7–14.2 μmol/L). The results of other laboratory tests, including a complete blood count/differential count, cholesterol profile, electrolytes, renal function parameters, serum IgG4 were within the normal range (Table 2). The Changes of tumor markers are summarized in Table 1. Overall, most tumor markers were within the normal range except high elevation of TPS and TPA. Abdominal ultrasonography demonstrated a 4 cm-sized hypoechoic mass in the pancreatic head with dilatation of the intrahepatic and extrahepatic bile ducts and the main pancreatic duct. The mass displayed hyper-enhancement in arterial phase and hypo-enhancement in the portal and delayed phase on CEUS, typical “fast-in and fast-out” contrast pattern of maligant tumor. Helical CT and MRI scan with contrast enhancement, revealed a nodule in the end of the dilated lower bile in the enlarged pancreatic head, with dilatation of the intrahepatic and extrahepatic bile ducts and the main pancreatic duct, stenosis or obliteration of the pancreatic portion of the common bile duct. The mass was hypoattenuated to the pancreas in the early phase, but attenuation increased in the delayed phase. Given the diagnosis of obstructive jaundice due to malignant pancreatic tumor, the patients choose palliative operative intervention of hepaticojejunostomy. 1 month after recovering and checking out, the patient was readmitted to our hospital complaining of jaundice recurrence. Laboratory data included: ALT of 498 IU/L (reference range, 9–60 IU/L), AST of 397 IU/L (reference range, 15–45 IU/L), GGT of 734 IU/L (reference range, 10–60 IU/L, ALP of 420 IU/L (reference range, 455–125 IU/L), Tbil of 119.6 μmol/L (reference range, 1.7–21 mg/dL), Dbil of 55.8 μmol/L (reference range, 0.0–6.8 μmol/L), and Ibil of 63.8 μmol/L (reference range, 1.7–14.2 μmol/L). MRI displayed an inflammatory infiltrate in the swollen pancreas, dilatation of the intrahepatic bile ducts and thicken edema wall of extrahepatic bile ducts, which was compatible with AIP. Further laboratory tests revealed the serum IgG4 was elevated to 266 mg/dL this time. The patient is skeptical for AIP and was given corticosteroid treatment as an outpatient and was well 11 months later. Since the operation, his serum IgG4 levels have decreased to be 40.3 (Table 2). Based on the postoperative recurrence of jaundice, elevated serum IgG4 level, MRI findings and good response to corticosteroid treatment, the diagnosis of AIP was then confirmed, although the specimen of bile cyst and regional lymph nodes showed no fibrous tissue with focal sclerotic stroma, focal lymphoid cell aggregation or IgG4-positive plasma cells.

## Discussion and conclusions

Since 2002, several diagnostic criteria for AIP and their amendments has been proposed by Asian countries (Japan and Korea) [4], USA (HISORt, Mayo Clinic) [5] and International Association of Pancreatology (International Consensus diagnostic criteria of Experts from Asia, North America, and Europe) [6]. With subtle inconsistency, clinical AIP diagnosis normally accord following consensus. (1): In the patients with typical AIP, US, CT and MRI were less likely to find a discrete pancreatic mass and more likely to find a diffusely enlarged pathological pancreatic head in a swollen pancreas [7]. ERCP and MRCP generally shows fairly distinctive irregular narrowing of the dilated main pancreatic duct [3]. However, focal form of AIP in the present study and PC share many features, including sudden jaundice, elevation of tumor markers, and imaging manifestations of focal mass in the pancreas, “double-duct sign” representing stricture in both the biliary and pancreatic ducts, and angiographic abnormalities, which make the discrimination very challenging. Also, the positive predictive value of radiological diagnosis for pancreatic cancer declines in patients with concurrent chronic pancreatitis [7]. (2): Typical cases also have markedly elevated serum IgG4 (serological cut-off > 135 mg/dL), hypergammaglobulinemia and a favorable response to steroid therapy [8–10]. However, the characteristic increased serum IgG4 level was not observed after operation in the first AIP case, which is confirmed by postoperative histological and immunohistochemical characteristics. In the second case, preoperative serological IgG4 are with normal range (98 mg/dL), which elevated to 266 mg/dL after surgery and decreased to 40.6 mg/dL after 3 months of corticosteroid treatment. Several studies report pancreatic masses in patients with particularly high serum IgG4 levels (> 135 mg/dL) histopathologically proven to be PC. Cases of the present study further declines the positive predictive value of elevated serum IgG4 levels (> 135 mg/dL) for AIP. The reason may be serum IgG4 level changes with the immune functional status, encompassing genetic predisposition, intrinsic and extrinsic triggers and their interaction with the immune system. The outcome of these processes lead to dynamic changes of activation of immune cells and cytokine levels, which in turn leads to spontaneously relapsing and remitting inflammatory injury in pancreas [11–13]. (3): Histological confirmation by biopsy and brush cytology, often guided by endoscopic ultrasound and ERCP respectively, could aid in making a correct diagnosis when there is uncertainty over the differentiation of PC from AIP. The major limitations of the technique are operator dependence and a limited field of visualization for detecting metastatic spread to the liver and peritoneum. Furthermore, there is a high rate of false-negative results as seen in a previous study, especially in patients who have the smallest lesions and/or in the presence of chronic pancreatitis, due to the inadequate sample provided (even by core biopsies) [3, 14]. In addition, as an autoimmune disease, the dynamic interaction between endogenous and exogenous factors and the immune system play a key role in the pathogenesis and propagation of inflammation as well as healing process, which may make preoperative histological test miss the histological and immunohistochemical characteristic of AIP. This may explain that the specimen of bile cyst and regional lymph nodes showed no fibrous tissue with focal sclerotic stroma, focal lymphoid cell aggregation or IgG4-positive plasma cells. Also, clinically, histologic diagnosis is not usually required for PC surgery to go ahead and most of PC-suspected patients cannot routinely have pre-operative histological confirmation to exclude relative rare untypical AIP. **(4):** Nonoperative management strategy with steroids are frequently used as diagnostic therapy of AIP [15]. However, with risk of delaying PC treatment, they are more suitable for following known AIP cases rather than preoperative differentiation diagnosis from PC. In all, considering the absence of preoperative clear-cut differential diagnostic tool and the fact of up to 10% of the PC-suspected patients undergoing unnecessary laparotomy for absence of typical characters of AIP [7] (just like both of the two cases here), extracting diagnostic value from routine presenting signs and symptoms is urgently needed to guide the clinician in the decision-making process.

**Fig. 1:**
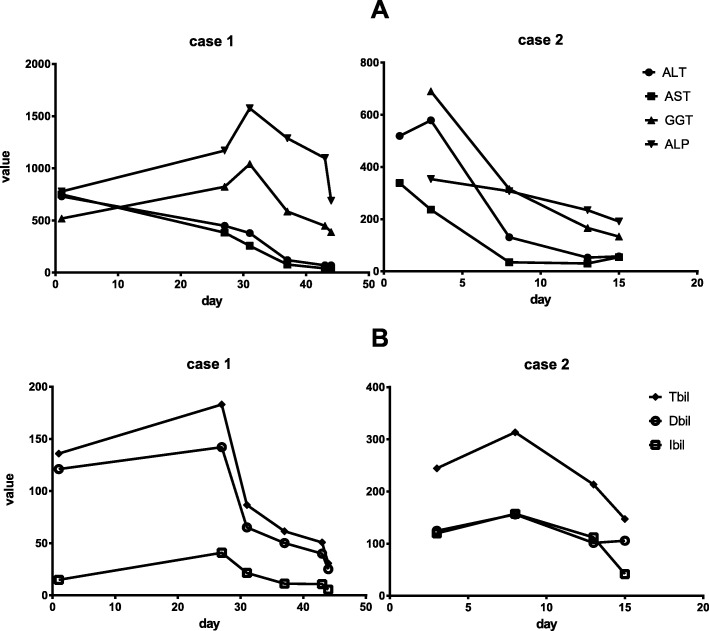
Intermittent liver damage presentation of liver enzymes including, AST, ALT, ALP, GGT (**a**) and mild raised but fluctuating cholestasis of Tbil, Dbil, Ibil (**b**) for both AIP cases

We extracted several characteristics that may help discriminate AIP from PC, through retrospectively analyzing work-up process of AIP in two patients who underwent laparotomy for suspected PC. (1): First, we found surprisingly that Liver function tests of both the two patients showed combination of results indicative of intermittent cholestasis and liver damage (mild raised but fluctuating levels of liver enzymes including Tbil, Dbil, Ibil, AST, ALT, ALP and GGT (Fig. 1). That intermittent jaundice which disappears spontaneously is obvious different to the progressive jaundice of PC and periampullary carcinoma, which represent highly elevated liver enzymes during short time [16, 17]. Contrary to the Hopkins and Mayo experience, patients with AIP from the present study were jaundiced less severe and consequently were less likely to undergo pre-operative biliary drainage procedures, comparable to the report of Bledsoe [10]. (2): In contrast to the young age of both the AIP patients (34 and 49 years old) here, 80% of cases of PC occur in those over 60 years old, and rarely occurs before the age of 40 [16, 17]. So, young age may help differentiate AIP from PC, especially for AIP type 2. (3): Tumor marker is a group of biomarkers found in blood that can be elevated by the presence of one or more types of cancer, each indicative of the presence of particular cancer. Tumor marker of CA19–9, CA 242 are frequently elevated in pancreatic cancer. However, with sensitivity of 80% and specificity of 73%, they are used more for following known cases rather than diagnosis [18]. In the first case, tumor marker of CA50, CA19–9, CA 242, TPS and TPA was highly elevated, while in the second case, postoperative serum levels of tumor markers TPA and TPS were elevated (Table 1). Overall, both of AIP cases has elevated TPS and TPA, which rarely occurs in PC. The reason is unknown, but contrary to the utilization of serum tissue polypeptide specific antigen (TPS) and tissue polypeptide antigen (TPA) as complements to CA 19–9 in the detection of pancreatic carcinoma in the last 4 decades [19–21]. (4): Agreement concerning mass location, vascular involvement, pathological lymph nodes among morphologic imagings of US, CT, EUS, MRI, PET-CT were significantly higher in PC than in AIP [3]. That was also noted in the present report: for the first case, in contrast to mass in the pancreatic head by esophagogastroduodenoscopy, PET-CT and MRI, abdominal ultrasonography showed a tumor in the uncinate process of the pancreas while enhanced computed tomography (CT) revealed a mass in the end of lower bile duct. For the second case, abdominal CEUS demonstrated a malignant mass in the pancreatic head, whereas Helical CT and MRI scan with contrast enhancement, revealed a nodule in the end of the dilated lower bile in the enlarged pancreatic head. In all, for both of the cases, the result of imaging modality cannot recapitulated those seen in the other ones. That was different to PC, whose similar observations by one imaging modality can usually be corroborated by other ones. The reason may be due to that the morphology of the mass and pancreas changes with the dynamic immune functional status [11–13]. (5): no obesity and new-onset diabetes mellitus. There is congruent data to support the association between PC development and obesity, new-onset diabetes mellitus (DM). Large epidemiologic and cohort studies have identified obesity and new-onset DM as high-risk factors for early detection of PC [22, 23]. For AIP, however, only 21% of patients developed diabetes mellitus (pancreatic endocrine insufficiency), of whom 73% required insulin [24]. In total, pancreatic endocrine insufficiency exist rather as long-term outcome than risk factor in patients with AIP, which may explain both of the AIP cases has no DM and obesity in the present study. (6): Comparable to this study, patients with AIP complained significantly more of pain as first presenting symptom, whereas more patients complained painless jaundice in the PC group [1, 10]. In all, patients with AIP presented more often with pain and less often with jaundice. (7):Also, patients with AIP tended to smoke significantly more often [3, 10], contrary to the non-smoking habit of both cases from the present study. Research gaps and opportunities to address the interplay and underlying mechanisms between smoking and AIP, PC need be outlined in the future work.

## Conclusions

In all, the clinical, laboratory and imaging presentation of patients with AIP and PC was very similar. Rather than relying on the rare typical signs, diagnosis of untypical AIP is probably best initiated by a panel of routine-check signs. We propose the combining absence or presence of above routine signs should make the clinician pay more attention to AIP diagnosis in PC-suspected patients in the decision-making process.

## Data Availability

The datasets generated during and/or analysed during the current study are available from the corresponding author on reasonable request.

## Abbreviations

AIP: autoimmune pancreatitis
IFL: indirect immunofluorescence
IgG4: immunoglobulin G4
ITM: immunoturbidimetry
PC: pancreatic cancer
WB: Western blotting

## References

[1] Matsumori T, Shiokawa M, Kodama Y (2018). Pancreatic mass in a patient with an increased serum level of IgG4. Gastroenterology..

[2] Bernasconi L, Mundwiler E, Regenass S, Aubert V, Hammerer-Lercher A, Heijnen I. Variable and inaccurate serum IgG4 levels resulting from lack of standardization in IgG subclass assay calibration. Clin Chem Lab Med. 2019. 10.1515/cclm-2019-0261 31188751

[3] Hsu WL, Chang SM, Wu PY, Chang CC (2018). Localized autoimmune pancreatitis mimicking pancreatic cancer: case report and literature review. J Int Med Res.

[4] Otsuki M, Chung JB, Okazaki K (2008). Asian diagnostic criteria for autoimmune pancreatitis: consensus of the Japan-Korea symposium on autoimmune pancreatitis. J Gastroenterol.

[5] Chari ST, Takahashi N, Levy MJ (2009). A diagnostic strategy to distinguish autoimmune pancreatitis from pancreatic cancer. Clin Gastroenterol Hepatol.

[6] Khosroshahi A, Wallace ZS, Crowe JL (2015). International consensus guidance statement on the management and treatment of IgG4-related disease. Arthritis Rheum.

[7] de Castro SM, de Nes LC, Nio CY (2010). Incidence and characteristics of chronic and lymphoplasmacytic sclerosing pancreatitis in patients scheduled to undergo a pancreatoduodenectomy. HPB (Oxford).

[8] Adachi K, Hashimoto K, Nonaka R (2017). A case of an IgG4-related inflammatory Pseudotumor of the liver showing enlargement that was difficult to differentiate from hepatic Cancer. Gan To Kagaku Ryoho.

[9] Hamano H, Tanaka E, Ishizaka N, Kawa S (2018). IgG4-related disease - a systemic disease that deserves attention regardless of One's subspecialty. Intern Med.

[10] Bledsoe JR, Della-Torre E, Rovati L, Deshpande V (2018). IgG4-related disease: review of the histopathologic features, differential diagnosis, and therapeutic approach. APMIS..

[11] Kubo S, Nakayamada S, Tanaka Y. Immunophenotype involved in IgG4-related disease. Mod Rheumatol. 2018:1–13. 10.1080/14397595.2018.1537962 30334637

[12] Hiratsuka I, Yamada H, Itoh M, et al. Changes in serum immunoglobulin G4 levels in patients with newly diagnosed Graves’ disease. Exp Clin Endocrinol Diabetes. 2018. 10.1055/a-0669-9333 30235492

[13] Konno N, Sugimoto M, Takagi T (2018). Changes in N-glycans of IgG4 and its relationship with the existence of hypocomplementemia and individual organ involvement in patients with IgG4-related disease. PLoS One.

[14] Bhattacharya A, Cruise M, Chahal P (2018). Endoscopic ultrasound guided 22 gauge core needle biopsy for the diagnosis of autoimmune pancreatitis. Pancreatology..

[15] Moon SH, Kim MH, Park DH (2008). Is a 2-week steroid trial after initial negative investigation for malignancy useful in differentiating autoimmune pancreatitis from pancreatic cancer? A prospective outcome study. Gut..

[16] Danai LV, Babic A, Rosenthal MH (2018). Altered exocrine function can drive adipose wasting in early pancreatic cancer. Nature..

[17] Ryan DP, Hong TS, Bardeesy N (2014). Pancreatic adenocarcinoma. N Engl J Med.

[18] Balachandran VP, Łuksza M, Zhao JN (2017). Identification of unique neoantigen qualities in long-term survivors of pancreatic cancer. Nature..

[19] Slesak B, Harlozinska-Szmyrka A, Knast W, Sedlaczek P, van Dalen A, Einarsson R (2000). Tissue polypeptide specific antigen (TPS), a marker for differentiation between pancreatic carcinoma and chronic pancreatitis. A comparative study with CA 19-9. Cancer..

[20] Talar-Wojnarowska R, Gasiorowska A, Olakowski M, Lekstan A, Lampe P, Malecka-Panas E (2010). Clinical value of serum neopterin, tissue polypeptide-specific antigen and CA19-9 levels in differential diagnosis between pancreatic cancer and chronic pancreatitis. Pancreatology..

[21] Basso D, Fabris C, Del FG (1988). Combined determination of serum CA 19-9 and tissue polypeptide antigen: why no improvement in pancreatic cancer diagnosis. Oncology..

[22] Tirkes T, Shah ZK, Takahashi N (2019). Reporting standards for chronic pancreatitis by using CT, MRI, and MR Cholangiopancreatography: the consortium for the study of chronic pancreatitis, diabetes, and pancreatic Cancer. Radiology..

[23] Tu J, Yang Y, Zhang J (2018). Effect of the disease severity on the risk of developing new-onset diabetes after acute pancreatitis. Medicine (Baltimore).

[24] Vujasinovic M, Valente R, Maier P (2018). Diagnosis, treatment and long-term outcome of autoimmune pancreatitis in Sweden. Pancreatology..

